# Effects on Cognitive Functioning of Acute, Subacute and Repeated Exposures to High Altitude

**DOI:** 10.3389/fphys.2018.01131

**Published:** 2018-08-21

**Authors:** Matiram Pun, Veronica Guadagni, Kaitlyn M. Bettauer, Lauren L. Drogos, Julie Aitken, Sara E. Hartmann, Michael Furian, Lara Muralt, Mona Lichtblau, Patrick R. Bader, Jean M. Rawling, Andrea B. Protzner, Silvia Ulrich, Konrad E. Bloch, Barry Giesbrecht, Marc J. Poulin

**Affiliations:** ^1^Department of Physiology & Pharmacology, Cumming School of Medicine, University of Calgary, Calgary, AB, Canada; ^2^Hotchkiss Brain Institute, Cumming School of Medicine, University of Calgary, Calgary, AB, Canada; ^3^Department of Clinical Neuroscience, Cumming School of Medicine, University of Calgary, Calgary, AB, Canada; ^4^O'Brien Institute for Public Health, Cumming School of Medicine, University of Calgary, Calgary, AB, Canada; ^5^Department of Psychology, Faculty of Science, University of British Columbia, Vancouver, BC, Canada; ^6^Biomedical Engineering Graduate Program, University of Calgary, Calgary, AB, Canada; ^7^Pulmonary Division, Sleep Disorders Centre and Pulmonary Hypertension Clinic, University Hospital Zürich, Zurich, Switzerland; ^8^Department of Family Medicine, Cumming School of Medicine, University of Calgary, Calgary, AB, Canada; ^9^Department of Psychology, Faculty of Arts, University of Calgary, Calgary, AB, Canada; ^10^Department of Psychological and Brain Sciences, Institute for Collaborative Biotechnologies, University of California, Santa Barbara, Santa Barbara, CA, United States; ^11^Libin Cardiovascular Institute of Alberta, Cumming School of Medicine, University of Calgary, Calgary, AB, Canada; ^12^Faculty of Kinesiology, University of Calgary, Calgary, AB, Canada

**Keywords:** altitude, cognition, hypoxia, brain, CANTAB, AMS/LLS, SpO_2_, ALMA

## Abstract

**Objective:** Neurocognitive functions are affected by high altitude, however the altitude effects of acclimatization and repeated exposures are unclear. We investigated the effects of acute, subacute and repeated exposure to 5,050 m on cognition among altitude-naïve participants compared to control subjects tested at low altitude.

**Methods:** Twenty-one altitude-naïve individuals (25.3 ± 3.8 years, 13 females) were exposed to 5,050 m for 1 week (*Cycle 1)* and re-exposed after a week of rest at sea-level (*Cycle 2*). Baseline (BL, 520 m), acute (Day 1, HA1) and acclimatization (Day 6, HA6, 5,050 m) measurements were taken in both cycles. Seventeen control subjects (24.9 ± 2.6 years, 12 females) were tested over a similar period in Calgary, Canada (1,103 m). The Reaction Time (RTI), Attention Switching Task (AST), Rapid Visual Processing (RVP) and One Touch Stockings of Cambridge (OTS) tasks were administered and outcomes were expressed in milliseconds/frequencies. Lake Louise Score (LLS) and blood oxygen saturation (SpO_2_) were recorded.

**Results:** In both cycles, no significant changes were found with acute exposure on the AST total score, mean latency and SD. Significant changes were found upon acclimatization solely in the altitude group, with improved AST Mean Latency [HA1 (588 ± 92) vs. HA6 (526 ± 91), *p* < 0.001] and Latency SD [HA1 (189 ± 86) vs. HA6 (135 ± 65), *p* < 0.001] compared to acute exposure, in *Cycle 1*. No significant differences were present in the control group. When entering Acute SpO_2_ (HA1-BL), Acclimatization SpO_2_ (HA6-BL) and LLS score as covariates for both cycles, the effects of acclimatization on AST outcomes disappeared indicating that the changes were partially explained by SpO_2_ and LLS. The changes in AST Mean Latency [ΔBL (−61.2 ± 70.2) vs. ΔHA6 (−28.0 ± 58), *p* = 0.005] and the changes in Latency SD [ΔBL (−28.4 ± 41.2) vs. ΔHA6 (−0.2235 ± 34.8), *p* = 0.007] across the two cycles were smaller with acclimatization. However, the percent changes did not differ between cycles. These results indicate independent effects of altitude across repeated exposures.

**Conclusions:** Selective and sustained attention are impaired at altitude and improves with acclimatization.The observed changes are associated, in part, with AMS score and SpO_2_. The gains in cognition with acclimatization during a first exposure are not carried over to repeated exposures.

## Introduction

Mountains occupy one fifth of the earth's surface and are popular destinations for a variety of activities such as trekking, climbing, pilgrimages, mining, scientific experiments and celestial observations. Further, more than 140 million people worldwide live at altitudes over >2,500 m, (Penaloza and Arias-Stella, 2007) and many high-altitude dwellers sojourn at lower altitudes. The barometric pressure decreases exponentially with altitude gain and this hypobaric hypoxia leads to reduced inspired partial pressure of oxygen (West, 1996). Unacclimatized lowlanders may suffer from cerebral symptoms such as headache, nausea, vomiting and impaired coordination when exposed to high altitudes (>2,500 m) (Hackett and Roach, 2001; Bärtsch and Swenson, 2013). The brain, particularly the hippocampus and other areas within the limbic system, is very sensitive and vulnerable to hypoxia (Hornbein, 2001; Virués-Ortega et al., 2004; Wilson et al., 2009).

High altitude exposure has a detrimental effect on cognitive functions with slower reaction times and reduced psychomotor vigilance i.e., slower reaction times as a measure of reduced sustained attention (high altitude, 1,500**–**3,500 m); impaired learning, spatial and working memory (very high altitude, 3,500–5,500 m) and impaired memory retrieval (extreme altitude, >5,500 m) (Virués-Ortega et al., 2004; Wilson et al., 2009; Yan, 2014; Taylor et al., 2016; Bickler et al., 2017; McMorris et al., 2017). The effects of hypoxia on cognitive functions have been previously explored among climbers (Kramer et al., 1993), trekkers (Dykiert et al., 2010), military personnel (Shukitt-Hale et al., 1998), flight crews (Nation et al., 2017), and high altitude residents (Virues-Ortega et al., 2011; Wang et al., 2014). Kramer et al. for example, report impairments in learning and memory processes especially when individuals were required to learn new skills while executing perceptual and semantic memory tasks. Similarly, Shukitt-Hale et al. report deterioration of both mood and performance in military personnel exposed to high-altitude. Futher, high altitude exposure has been shown to increase reaction times and impair memory encoding and retention (McMorris et al., 2017; Nation et al., 2017). However, Dykiert et al. suggest more pronounced changes in mean reaction times only above 4,000 m. Different types of hypoxic exposures such as field (Subudhi et al., 2014; Davranche et al., 2016), simulated hypobaric hypoxia (Hornbein et al., 1989; Asmaro et al., 2013; Malle et al., 2013), normobaric hypoxia (Turner et al., 2015) and intermittent hypoxia (Champod et al., 2013) have been investigated. Davranche et al. showed impaired information processing (speed and accuracy) at high altitude while Hornbein et al. reported impaired visual long-term memory during chamber simulation. With very high altitude exposure (5,260 m), Subudhi et al. found impairments in reaction times and in performance on the code substitution tasks (simultaneous and match to sample) which then improved with acclimatization. In the study by Malle et al., an increased rate of error frequency and worsened working memory were reported while Asmaro et al. observed impairments in cognitive flexibility and attention, short-term and working memory and executive functions. Similarly, Turner et al found that acute normobaric hypoxia affected memory, attention and executive functions. Although the aforementioned studies differ in types of hypoxic exposure, duration, modality and severity, the reported neurocognitive impairments are consistent across studies (Virués-Ortega et al., 2004; McMorris et al., 2017). Regardless, the significant differences across study design and the inadequately powered sample sizes limit the currently available studies and futher research is therefore needed.

A large number of high altitude workers, such as the ones involved in the large mining industry in the Chilean mountains or scientists, engineers and staff at the Atacama Large Millimeter/submillimeter Array (ALMA) scientific observatory, travel periodically to high altitudes for work. The workers at ALMA are periodically exposed to “very high altitude” (i.e., 5,050 m) for an entire week [including sleep periods at “high altitude” (i.e., 2,950 m)] followed by a week of rest at near-sea level (i.e., 520 m). Hypoxia associated with high altitude may impair cognitive performance and therefore it may lead to higher rates of errors (Hornbein et al., 1989; Davranche et al., 2016) and elevated risks of occupational injuries while performing their duties as seen among high altitude miners (Vearrier and Greenberg, 2011). However, there is no study examining the effects of this unique ascent profile and work schedule at very high altitude on cognitive functioning.

Hence, here we investigate the effects of acute, acclimatization, and repeated exposure to very high altitude on cognitive functions in altitude-naïve individuals bringing them to ALMA (5,050 m) with the same schedule that the workers would follow over a month. We hypothesize that acute exposure to high altitude will result in slower reaction times, decreased attention and reduced executive functions (reduced flexibility and ability to shift, greater fixation, reduced executive control and planning ability) which will then be restored with acclimatization. Further, we hypothesize that the positive changes in cognitive function due to acclimatization will be carried over to repeated exposure after a week of rest at low altitude. Finally, we aim to explore the role of AMS and blood oxygen saturation (SpO_2_) on changes in cognitive functions.

## Materials and methods

### Participants

**Figure 1:**
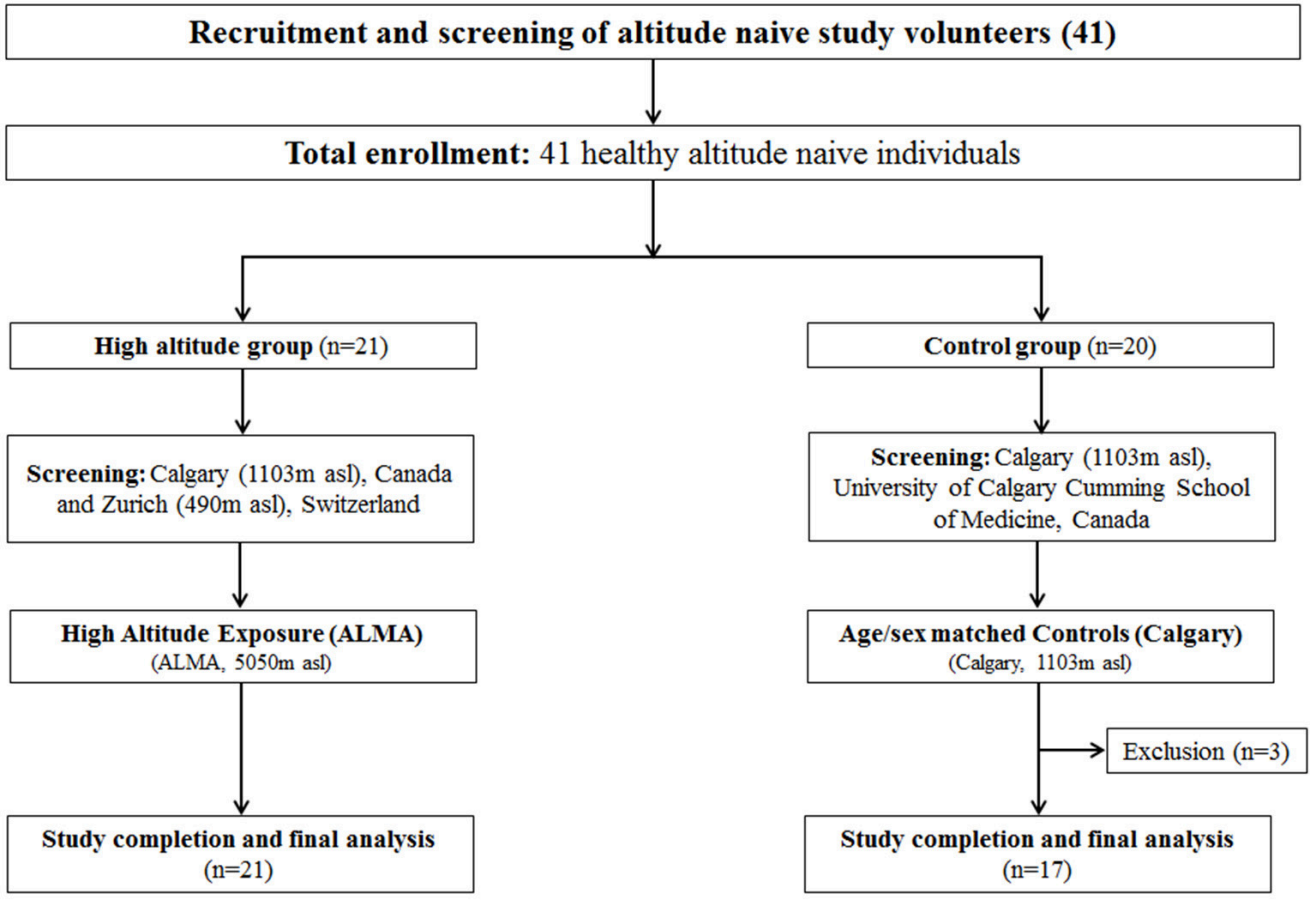
Study flowchart for Altitude and Control group study participants. Figure illustrates participant flow through the experimental protocols (CONSORT diagram) showing the flow of study participants for high altitude protocol (ALMA) and controls (Calgary). n, number; asl, above sea level; m, meter; kg, kilogram; ALMA, the Atacama Large Millimeter Array.

A total of 41 participants (21 altitude-exposed, 20 controls) were recruited. All participants provided written informed consent. Inclusion criteria were currently living at < 1,300 m (participants must have been living in Calgary, 1,103 m, for at least 1 year) and no overnight stays at altitudes >1,500 m during the 4 weeks preceding the study. Exclusion criteria included previous history of altitude illnesses at moderate altitude (< 3,000 m), current pregnancy, and health impairment that required regular treatment. The screening for the inclusion/exclusion of participants was carried out at the Foothills Medical Center, Cumming School of Medicine, University of Calgary, Calgary, Canada (1,103 m). Twenty-one altitude-naive healthy young adults (age = 25.2 ± 3.7 years, education = 17.1 ± 2.5 years, 13 females, BMI = 24.5 ± 8.1 kg·m^−2^) took part in the high-altitude expedition, 18 of whom lived in Calgary and three of whom lived in Zurich and surrounding area (Switzerland, altitude 490 m) and were therefore screened at University Hospital of Zurich, Zurich, Switzerland. Twenty altitude-naive healthy young adults completed the testing sessions at the University of Calgary, Canada (altitude, 1,103 m), and formed the control group. Within the control group, three participants did not complete the cognitive sessions for reasons external to the study, and therefore were excluded from the analyses. Hence, the control group included a total of 17 participants in the final analyses (age = 24.9 ± 2.6 years, education = 16.8 ± 1.8 years, 12 females, BMI = 23.4 ± 2.7 kgm^−2^). The total final sample included in the analyses (from both Altitude and Control) consisted of 38 participants (age = 25.1 ± 3.2 years, education = 17 ± 2.2 years, 25 females). The study was approved by the University of Calgary Conjoint Health Research Ethics Board (CHREB ID: REB15-2709) and registered as a clinical trial in ClinicalTrials.gov (NCT02738307). The flow of study participants through the Altitude and Control protocols is illustrated in Figure 1.

### Study design

**Figure 2:**
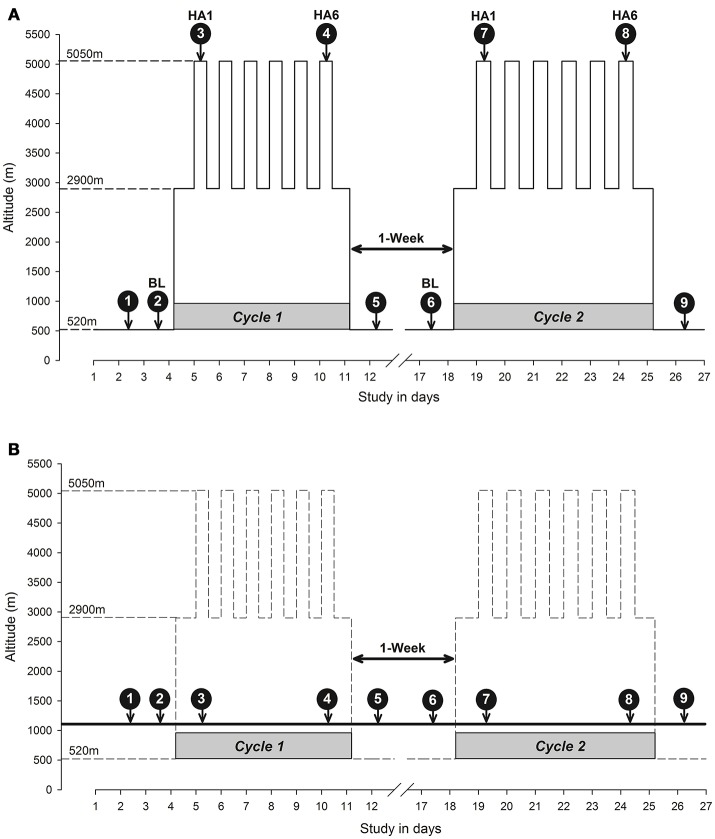
Study design diagram: Altitude vs. Control. **(A)** Altitude study protocol in which study participants were exposed to altitude at ALMA; **(B)** Control study protocol in which data were collected in Calgary. The y-axis depicts altitude in meters and the x-axis depicts the study time in days. The dashed lines connecting to the y-axis indicates altitude as baseline altitude (Santiago, 520 m), sleeping altitude (ALMA Operations Support Facility Center, 2,900 m) and high altitude working station (ALMA, 5,050 m). The downward arrows indicate the nine cognitive function testing sessions over 26 days at corresponding altitude in y-axis and expedition days in x-axis during two cycles of high altitude expedition interspersed with a week of resting at low altitude. The average altitude exposure at ALMA Observatory work station during each day was ~4–8 hrs/day. The remainder of the time was spent at the ALMA base camp of an altitude of 2,900 m to sleep. In the control protocol **(B)**, the high-altitude cycle (expedition) has been depicted as dashed lines but experimental altitude (Calgary, 1,103 m asl) has been depicted as a bold solid line crossing the two cycles with data collection time points (white numbers inside black filled circles with arrows going down to respective days matching high altitude protocol similar to **A**). The solid line with arrows on both sides between two cycles (1-Week) indicates 7 days rest a low altitude separating two cycles in both panels. BL, baseline; HA1, high altitude acute exposure at day 1; HA6, high altitude acclimatization exposure at day 6; 1-Week, one week break between two cycles of expedition in **(A)** and data collection in **(B)**.

The high-altitude exposure schedule spanned over the course of a month with two cycles of high altitude exposure (*Cycle 1* and *Cycle 2*) separated by a week at low altitude (Figure 2). The baseline measurements were taken in Santiago, Chile (520 m). The participants then flew to the Calama airport (~2 h) and took a bus (~2 h) to the basecamp at The Atacama Large Millimeter/submillimeter Array (ALMA) Operation Support Facility (ALMA ASF; 2,900 m). The participants traveled to the ALMA Observatory (5,050 m) by motor vehicle (about 45 min). Throughout the 6-day high altitude expedition (*Cycles 1* and *2*), participants spent nights at a support facility (ASF, 2,900 m) and commuted to ALMA Observatory (5,050 m) by motor vehicle to spend 4–8 h each day. According to ALMA policy, the participants were allowed to spend only 4 h at 5,050 m on the first day. Recovery measurements were taken in Santiago, Chile (520 m). The control subjects followed the same testing schedule as the altitude group at the Brain Dynamics Lab, The University of Calgary, Calgary, Canada without changes in altitude (1,103 m). An overview of the cognitive testing schedule is illustrated in Figure 2 (Figure 2A for altitude and Figure 2B for control). The first test was a familiarization test in *Cycle 1* while the second test was the baseline (BL). The first measurement at altitude (test 3 on day 1 of altitude exposure) was an acute exposure test (HA1) while test 4 at altitude (on day 6 of altitude exposure) was an acclimatization measurement (HA6). A similar schedule was followed in *Cycle 2*, and the same testing schedule and protocol (i.e., high-altitude protocol) was mirrored in the control group.

### Cognitive test battery

**Figure 3:**
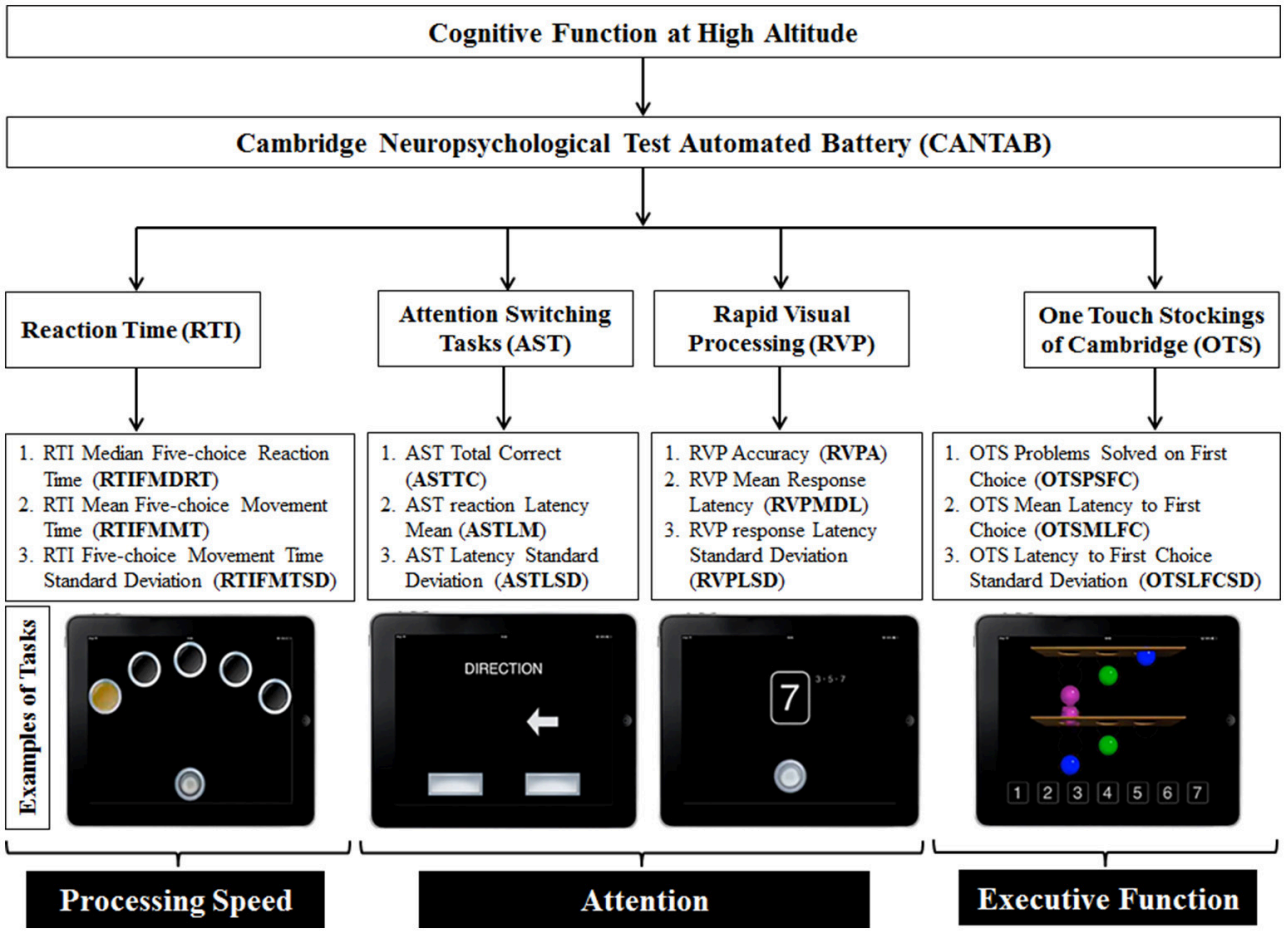
CANTAB battery tests that were incorporated in the study. Reaction time (RTI), attention (AST and RVP) and executive functions (OTS) have been tested. The red boxes indicate the grouping classification of the test batteries i.e. reaction time, attention and executive function. The bottom part of the figure illustrates representative example pictures of respective CANTAB battery/task used in the study (Cambridge Cognition, 1994) from left to right: RTI, AST, RVP, and OTS.

Cognitive tests assessed three domains of cognitive function: processing speed, sustained attention and executive functions. We created a custom battery with tests available within the Cambridge Neuropsychological Test Automated Battery (CANTAB® Cogntive Assessment Software; Cambridge Cognition, 1994). The CANTAB cognition battery can be administered several times while controlling for learning and repetition effects (Lowe and Rabbitt, 1998; Syväoja et al., 2015). This is achieved by generating random stimuli each time a participant logs in into his/her account for a new testing phase. The tests were administered on iPad Air 1 (model: A1474, dimensions: 9.7 inches retina display, iSO 9.3.1, Apple Inc., Cupertino, CA) and were completed in 30 min. The battery was administered nine times over the course of the expedition and nine times in controls as illustrated in Figure 2 (Figure 2A for altitude and Figure 2B for controls). The individual components of the CANTAB battery included the Reaction Time (RTI) task to assess processing speed, the Attention Switching Task (AST) and the Rapid Visual Processing (RVP) task to assess attention and the One Touch Stockings of Cambridge (OTS) to test executive functions. The CANTAB cognition outcome measures, along with their abbreviations, and examples of the tasks, have been illustrated in Figure 3.

The RTI task is a measure of motor and processing speed. Participants are required to hold a button at the bottom of the screen (starting position). Circles are presented at the top of the screen (either 1 or 5 circles) and at some point, one of the circles will flash yellow. Participants must then tap the highlighted circle as quickly as possible and then go back to the starting position. The outcome measures that we analyzed for this task are limited to the harder condition including 5 circles. The median reaction time (RTIFMDRT) considers how long it takes for the participants to touch the yellow circle after perceiving the change in color. Movement time (RTIFMMT) instead refers to the interval between the release of the starting position button and contact with the yellow circle. The AST measures individuals' ability to inhibit irrelevant information (selective attention). An arrow appears on the screen either on the left or right portion of the screen, pointing in either direction. Each trial displays a cue prompting the participant to press the left or right button on the screen to evaluate either the position of the arrow in the screen or the direction where the arrow is pointing. The RVP task is a measure of sustained attention. During the test, an array of numbers from 2 to 9 is presented in a pseudo-randomized order in the middle of the screen. The participants are required to press as fast as they can a button at the bottom of the screen when they see a certain array (2-4-6, 4-6-8, and 3-5-7). The OTS task measures executive function and the ability to plan. The participants are shown combinations of three-dimensional (3-D) colored balls and they must indicate in a box at the bottom of the screen, the number of moves (least possible amount) that they think are required to reproduce the same combination from a different starting position. All the CANTAB parameters were measured in milliseconds (ms) except ASTTC and OTSPSFC which represented frequencies (n).

### Lake louise score (LLS) and SpO_2_

AMS was assessed using the Lake Louise Score (LLS) (Roach et al., 1993) and diagnosed when the total LLS score was ≥5 (Maggiorini et al., 1998; Dellasanta et al., 2007). The SpO_2_ was measured at rest with a finger pulse oximeter placed on the index finger.

#### Data analyses

##### Cognitive outcomes

Cognitive changes over the course of the high-altitude exposure compared to baseline were analyzed with a series of Repeated Measures Mixed Model Analyses of Variance (RM-ANOVAs). We utilized RM-ANOVA to test our *a priori* hypothesis of expected changes in cognitive outcomes from baseline (BL, 520 m) to acute exposure to altitude (HA1) and acclimatization period (HA6) during each cycle. The cognitive outcome measures at BL, HA1 and HA6 were entered as within-subjects factors (altitude exposure^*^3) while group (Altitude vs. Control) as between-subjects factor in the analysis model. Cognitive outcome measures (RTI, AST, RVP and OTS) for *Cycle 1* and *Cycle 2* were analyzed separately to test the specific hypothesis of carry-over effects due to re exposure to very high altitude. All the analyses were two-tailed, and statistical significance was set at *p* < 0.05. Descriptive data were expressed as mean ± standard deviation (mean ± SD) and were assessed for violation of normality. Greenhouse-Geisser (GG) correction was used when sphericity, as measured with the Mauchly's test, was violated. Only significant altitude exposure (BL, HA1, HA6) ^*^ group (altitude, controls) interactions were followed up by using pairwise comparisons with Bonferroni corrections to test within group differencies at each altitude exposure. The statistical analyses were carried out using the Statistical Package for the Social Sciences (SPSS, Version 24, IBM Corporation, New York 10504-1722, USA). The graphical illustration (study design and changes in cognition plots) were genereated using SyStat (SigmaPlot 13.0, Systat Software Inc, San Jose, CA, USA).

##### Covariates

To examine the contribution of SpO_2_ changes and total LLS to changes in cognitive measure due to altitude exposure, we used a Repeated Measures Analyses of Covariance (RM-ANCOVAs) with either SpO_2_ changes or LLS as covariates. The changes in SpO_2_ with altitude exposure were computed as acute change in SpO_2_ (Acute SpO_2_ = HA1 – BL), acclimatization change in SpO_2_ (Acclimatization SpO_2_ = HA6 – BL) and change in SpO_2_ at high altitude (Altitude SpO_2_ = HA6 – HA1). The changes were calculated for both cycles separately.

##### Acclimatization carry-over effects over the cycles

We tested the carry-over effects from *Cycle 1* to *Cycle 2* by computing differences in scores at baseline (ΔBL), acute exposure (ΔHA1) and acclimatization (ΔHA6) for each cognitive outcome measures i.e., change (Δ) = *Cycle 2* – *Cycle 1* at each data point in two cycles. Another RM-ANOVA was used to analyze persisting differences across the two cycles at different altitude exposures. We further extended our analysis to explore the acclimatization carry-over effects by calculating percent change in the cognitive variables and comparing them between the two cycles with paired t-tests. The acute percent change was calculated as [(BL-HA1)/BL^*^100] and acclimatization percent change as [(BL-HA6)/BL^*^100] whereas percent change at high altitude as [(HA1-HA6)/HA1^*^100] for both cycles.

## Results

Details of the analyzed cognitive outcomes (mean ± SD) with RM-ANOVA for the altitude group and control participants are presented in Table 1. The more extensive descriptive data for all valid entries across all the sessions for both *Cycle 1* (Familiarization, FL; Baseline, BL; Acute exposure, HA1 and Acclimatization exposure, HA6 and Recovery, REC) and *Cycle 2* (Baseline, BL; Acute exposure, HA1 and Acclimatization exposure, HA6 and Recovery, REC) in both altitude and control groups have been presented in the Supplemental Table 1.

**Table 1:** Cognitive parameters of altitude and control participants at baseline, acute and acclimatization exposures from *Cycle 1* and *Cycle 2*.

	***Cycle 1***						***Cycle 2***					
	**BL**		**HA1**		**HA6**		**BL**		**HA1**		**HA6**	
	**Alt (Sant)**	**Ctrl (Calg)**	**Alt (ALMA)**	**Ctrl (Calg)**	**Alt (ALMA)**	**Ctrl (Calg)**	**Alt (Sant)**	**Ctrl (Calg)**	**Alt (ALMA)**	**Ctrl (Calg)**	**Alt (ALMA)**	**Ctrl (Calg)**
**REACTION TIME (ms)**												
RTIFMDRT	365.3 ± 40.2	356.8 ± 34.5	379.4 ± 54.4	354.9 ± 49.1	355.0 ± 33.8	352.8 ± 30.8	362.7 ± 38.5	354.9 ± 32.9	358.5 ± 33.3	350.2 ± 29.4	351.0 ± 41.6	348.7 ± 26.5
RTIFMMT	192.4 ± 76.0	189.9 ± 44.1	242.3 ± 145.2	188.6 ± 43.3	203.5 ± 78.3	190.5 ± 41.5	198.0 ± 85.3	187.5 ± 38.9	203.5 ± 74.6	185.4 ± 39.5	175.8 ± 61.9	184.9 ± 41.0
RTIFMTSD	26.5 ± 10.3	31.3 ± 11.3	48.3 ± 44.0	38.7 ± 22.2	27.7 ± 11.8	34.1 ± 17.8	30.6 ± 15.6	31.9 ± 16.8	35.3 ± 21.2	27.7 ± 11.7	23.1 ± 7.5	36.3 ± 27.4
**ATTENTION (ms/*****n*****)**												
ASTTC *(n)*	155.3 ± 7.7	157.8 ± 2.7	151.9 ± 13.2	157.2 ± 2.6	156.4 ± 3.5	157.4 ± 2.3	156.0 ± 3.0	158.0 ± 2.5	152.1 ± 7.3	157.1 ± 2.5	154.8 ± 3.7	156.9 ± 3.3
ASTLM	572.6 ± 98.1	516.0 ± 83.6	574.9 ± 84.3^***^	494.6 ± 77.0	517.1 ± 81.4^***^	490.8 ± 78.8	510.1 ± 77.7	469.9 ± 67.8	528.4 ± 96.6^*^	471.9 ± 70.2	485.2 ± 82.1^*^	470.7 ± 63.9
ASTLSD	166.3 ± 61.9	131.4 ± 62.9	177.0 ± 78.9^***^	127.1 ± 46.9	125.8 ± 57.5^***^	120.6 ± 56.0	137.3 ± 62.0	107.1 ± 41.0	164.3 ± 82.3^**^	113.1 ± 43.2	122.5 ± 53.4^**^	115.6 ± 49.0
RVPA	0.96 ± 0.05	0.96 ± 0.07	0.94 ± 0.08	0.97 ± 0.03	0.97 ± 0.04	0.98 ± 0.02	0.97 ± 0.05	0.98 ± 0.02	0.96 ± 0.05	0.99 ± 0.02	0.98 ± 0.04	0.99 ± 0.02
RVPMDL	427.0 ± 41.1	402.9 ± 50.0	467.0 ± 110.7	395.2 ± 44.2	410.1 ± 35.2	391.4 ± 35.1	414.7 ± 46.4	381.5 ± 34.7	414.8 ± 34.8	378.6 ± 34.6	392.9 ± 36.6	394.6 ± 42.2
RVPLSD	109.5 ± 43.0	117.8 ± 123.0	123.0 ± 91.6	95.8 ± 63.4	104.1 ± 67.9	91.4 ± 47.0	103.4 ± 58.3	81.2 ± 44.0	132.7 ± 95.1	85.3 ± 40.0	88.8 ± 44.7	85.6 ± 56.6
**EXECUTIVE FUNCTION (ms/*****n*****)**												
OTSPSFC *(n)*	12.4 ± 1.8	12.2 ± 1.8	11.9 ± 1.7	12.5 ± 0.9	12.1 ± 2.0	12.4 ± 1.5	13.1 ± 1.6	12.8 ± 1.9	12.0 ± 2.0	12.9 ± 1.9	12.6 ± 1.7	12.8 ± 1.6
OTSMLFC	*14, 575.5*±*5, 670.1*	*13, 615.5*±*5, 282.7*	*11, 917.9*±*5, 382.6*	*14, 092.9*±*5, 766.3*	*10, 960.2*±*7, 442.5*	*10, 650.0*±*4, 035.9*	*11, 724.9*±*14, 035.0*	*10, 190.8*±*4, 799.3*	*9, 014.3*±*5, 038.3*	*10, 188.3*±*4, 683.9*	*6, 940*±*2, 141*	*9, 711.0*±*3, 933.1*
OTSLFCSD	*16, 425.9*±*9, 036.1*	*14, 867.7*±*8, 546.1*	*12, 687.6*±*7, 740.9*	*15, 925.1*±*10, 273.4*	*10, 531.6*±*9, 235.8*	*11, 074.2*±*6, 830.9*	*13, 783.4*±*20, 937.9*	*10, 490.0*±*7, 068.4*	*9, 544.1*±*7, 884.5*	*11, 474.9*±*7, 297.5*	*5, 990.2*±*2, 673.6*	*11, 422.2*±*7, 297.2*

*The mean and SD presented here are from the Repeated Measure ANOVA*.

*** *< 0.001*,

** *< 0.01*,

* <0.05; The asterisks (^*^) indicate statistically significant group-by-altitude exposure interaction.

### High altitude exposure: *cycle 1*

#### Cognitive outcomes

##### Reaction time (RTI)

There was a significant main effect of altitude exposure (BL = 361.0 ± 37.0, HA1 = 367.1 ± 52.5, HA6 = 353.8 ± 31.8) on the RTIFMTSD [*F*_(1.331, 42.590)_ = 6.01, *p* = 0.012 _GG_, $\eta_{p}^{2}$ = 0.158). However, there was no group-by-phase interaction (*p* = 0.181) meaning that there were no differences between the altitude group and controls at different altitude exposures. There was no main effect of altitude or group-by-phase interaction on the RTIFMDRT and the RTIFMMT.

##### Attention switching task (AST)

There was a main effect of altitude exposure [BL = 544.3 ± 94.2, HA1 = 534.7 ± 89.3, HA6 = 503.9 ± 79.9, *F*_(2, 64)_ = 11.2*, p* < 0.001, $\eta_{p}^{2}$ = 0.259] and a group by altitude exposure interaction (altitude vs. controls) [*F*_(2, 64)_ = 4.6*, p* = 0.013, $\eta_{p}^{2}$ = 0.126] on the ASTLM scores. This means that there were significant differences between the altitude and control groups at different altitudes. Follow-up pairwise comparisons revealed indeed that, in the altitude group, the ASTLM score was not impacted by acute exposure to altitude, but decreased with the acclimatization compared to acute exposure [HA1 (587.9 ± 91.9) vs. HA6 (525.8 ± 91.2), *t*_(19)_ = 5.784*, p* < 0.001]. No significant differences were present in the control group. We observed similar effects for the ASTLSD with a main effect of altitude exposure [BL = 148.8 ± 63.9, HA1 = 152.0 ± 69.0, HA6 = 123.2 ± 55.9, *F*_(2, 64)_ = 9.3, *p* < 0.001, $\eta_{p}^{2}$ = 0.226] and a group (altitude *vs* controls) by altitude exposure interaction [*F*_(2, 64)_ = 4.8*, p* = 0.011, $\eta_{p}^{2}$ = 0.131]. At follow up comparisons in the altitude group exclusively, the ASTLSD score was not impacted by acute exposure to altitude but decreased with the acclimatization as compared to acute exposure [HA1 (189.4 ± 86.1) vs. HA6 (134.5 ± 64.9); *t*_(19)_ = 5.427*, p* < 0.001]. No significant differences were found in the control group. There was no main effect of altitude exposure, nor group (altitude vs. control) by altitude exposure interaction for the ASTTC score.

##### Rapid visual processing (RVP)

There was a significant main effect of altitude exposure on the RVPA score [BL = 0.95 ± 0.05, HA1 = 0.95 ± 0.06, HA6 = 0.97 ± 0.03; *F*_(2, 64)_ = 3.5, *p* = 0.037, $\eta_{p}^{2}$ = 0.098] and on the RVPMDL score [BL = 414.9 ± 46.7, HA1 = 431.1 ± 90.6, HA6 = 400.7 ± 35.8; *F*_(1.376, 44.021)_ = 3.6, *p* = 0.03, $\eta_{p}^{2}$ = 0.1]. However, the group by altitude exposure interactions showed only a trend (RVPA, *p* = 0.162, $\eta_{p}^{2}$ = 0.055 and RVPMDL, *p* = 0.064, $\eta_{p}^{2}$ = 0.093). The RVPLSD did not change with altitude or between groups.

##### One touch stockings of cambridge (OTS)

We observed a main effect of altitude exposure on both OTSMLFC [BL = 12.26 ± 1.78, HA1 = 12.20 ± 1.38, HA6 = 12.24 ± 1.74; *F*_(2, 64)_ = 6.861, *p* = 0.002, $\eta_{p}^{2}$ = 0.177] score and OTSLFCSD [BL = 15646.7 ± 8696.2, HA1 = 14306.3 ± 9106.2, HA6 = 10802.8 ± 8003.5; *F*_(2, 64)_ = 5.081, *p* = 0.009, $\eta_{p}^{2}$ = 0.137] but no group by altitude exposure interactions. However, there was no main effect of altitude exposure and group by altitude exposure interaction on the OTSPSFC score.

#### SpO_2_ and lake louise score (LLS)

The effect of altitude exposure on ASTLM reported previously during acute and acclimatization visits disappeared when controlling for Acute SpO_2_ and Acclimatization SpO_2_. However, the effect of altitude exposure on ASTLM persisted when controlling for Altitude SpO_2_ [*F*_(2, 30)_ = 7.6*, p* = 0.002, $\eta_{p}^{2}$ = 0.338]. Similar observations were found in ASTLSD. The differences in altitude exposure on ASTLSD when controlling for both Acute SpO_2_ and Acclimatization SpO_2_ disappeared but the altitude exposure effect persisted when controlling for Altitude SpO_2_ [*F*_(2, 30)_ = 9.2, *p* = 0.001, $\eta_{p}^{2}$ = 0.38]. During acute exposure (HA1), when total LLS was entered as covariate, the changes in ASTLM due to altitude still persisted [*F*_(2, 30)_ = 3.6, *p* = 0.039, $\eta_{p}^{2}$ = 0.195]. On the contrary, the effect of altitude exposure on ASTLSD disappeared with LLS as covariate.

### High altitude exposure: *cycle 2*

#### Cognitive outcomes

##### Reaction time (RTI)

The RTIFMTSD score had no main effect of altitude exposure, but a group by altitude exposure interaction [*F*_(2, 70)_ = 5.96*, p* = 0.004, $\eta_{p}^{2}$ = 0.145]. The follow up comparisons showed that the RTIFMTSD score was not affected by the acute altitude exposure. However, it decreased after the acclimatization from acute exposure [HA1 (35.2 ± 21.1) vs. HA6 (23 ± 8), *t*_(19)_ = 2.590*, p* = 0.018]. These results, however, did not survive Bonferroni correction for multiple comparisons. The RM-ANOVA on the RTIFMDRT score and on the RTIFMMT score did not show any significant effects of altitude exposure. No significant differences were present in the control group.

##### Attention switching task (AST)

There was a main effect of altitude exposure [BL = 156.8 ± 2.9, HA1 = 154 ± 6, HA6 = 155.7 ± 3.6, *F*_(1.436, 50.272)_ = 7.6, *p* = 0.004_GG_, $\eta_{p}^{2}$ = 0.178] on the ASTTC and a group by altitude exposure interaction [*F*_(1.436, 50.272)_ = 3.6, *p* = 0.034_GG_, $\eta_{p}^{2}$ = 0.092]. In the follow up comparisons, the ASTTC decreased from baseline to altitude [BL (156 ± 3) vs. HA1 (152.3 ± 7.2), *t*_(20)_ = 2.889, *p* = 0.009] and improved with the acclimatization [HA1 (152.1 ± 7.2) vs. HA6 (154.7 ± 3.6), *t*_(19)_ = −2.309*, p* = 0.032] only in the altitude group. However, the changes did not survive multiple comparisons correction. There was a main effect of altitude exposure (BL = 492 ± 75, HA1 = 502 ± 89, HA6 = 478.5 ± 73.6, *F*_(2, 70)_ = 3.471*, p* = 0.037, $\eta_{p}^{2}$ = 0.09] on the ASTLM score and a group by altitude exposure interaction [*F*_(2, 70)_ = 3.144*, p* = 0.049, $\eta_{p}^{2}$ = 0.082]. In the follow-up comparisons, ASTLM score slightly increased with acute exposure although this change was not significant. However, ASTLM score significantly decreased with the acclimatization compared to acute exposure [HA1 (528.4 ± 96.6) vs. HA6 (485.2 ± 82.1), *t*_(19)_ = 2.879*, p* = 0.010] in the altitude group and not in the control group. Similarly, we found a main effect of altitude exposure (BL = 123.4 ± 54.8, HA1 = 140.7 ± 71.2, HA6 = 119.3 ± 50.8, *F*_(2, 70)_ = 3.6, *p* = 0.032, $\eta_{p}^{2}$ = 0.093] on the ASTLSD score, and a group by altitude exposure interaction [*F*_(2, 70)_ = 4.0*, p* = 0.023, $\eta_{p}^{2}$ = 0.103]. On follow-up comparisons, difference in ASTLSD score was only a trend [BL (135 ± 61) vs. HA1 (161.1 ± 81.5), *t*_(20)_ = −1.845*, p* = 0.080]. ASTLSD decreased with the acclimatization compared to acute exposure [HA1 (164.3 ± 82.3) vs. HA6 (122.4 ± 53.3), *t*_(19)_ = 3.005, *p* = 0.007]. The AST variables did not change significantly over different time points in the control group as we observed in the altitude group.

##### Rapid visual processing (RVP)

There was no main effect of altitude exposure nor group by altitude exposure interaction on the RVPA. There was no main effect of altitude exposure on the RVPMDL score, but there was a group by altitude exposure interaction [*F*_(2, 70)_ = 4.7*, p* = 0.012, $\eta_{p}^{2}$ = 0.119]. However, when directly compared, there was no difference. There was a trend to decrease with acclimatization compared to acute exposure [HA1 (414.8 ± 34.7) vs. HA6 (392.8 ± 36.5), *t*_(19)_ = 2.081*, p* = 0.051] on RVPMDL, however this trend did not survive a correction for multiple comparisons (Bonferroni). The RVPLSD score did not change over time with the exposure to altitude. The RVP variables in control group did not vary significantly over time.

##### One touch stockings of cambridge (OTS)

There were no significant main effects nor interactions on the OTSPSFC score, OTSMLFC and OTSLFCSD.

#### SpO_2_ and lake louise score (LLS)

The changes in SpO_2_ for *Cycle 2* were calculated in a similar manner as in *Cycle 1* and entered as covariates in the RM-ANCOVA analysis to investigate the role of ΔSpO_2_ cognitive changes at altitude. The significant changes due to altitude exposure persisted when controlling for Altitude SpO_2_ [*F*_(2, 30)_ = 3.6, *p* = 0.037, $\eta_{p}^{2}$ = 0.167] on the ASTLM score but disappeared when controlling for Acute ΔSpO_2_ and Acclimatization SpO_2_. The significant changes on the ASTLSD score due to altitude exposure persisted even after controlling for Altitude SpO_2_ [*F*_(2, 30)_ = 3.7, *p* = 0.033, $\eta_{p}^{2}$ = 0.172] but there were no significant changes when controlling for Acute SpO_2_ and Acclimatization SpO_2_. The total LLS was entered as covariate in the ANCOVA to investigate the role of AMS symptoms on the ASTLM and ASTLSD scores relative to HA1. The cognitive changes related to different exposures to altitude disappeared with AMS score as covariate.

### Acclimatization carry-over effects over the cycles

**Figure 4:**
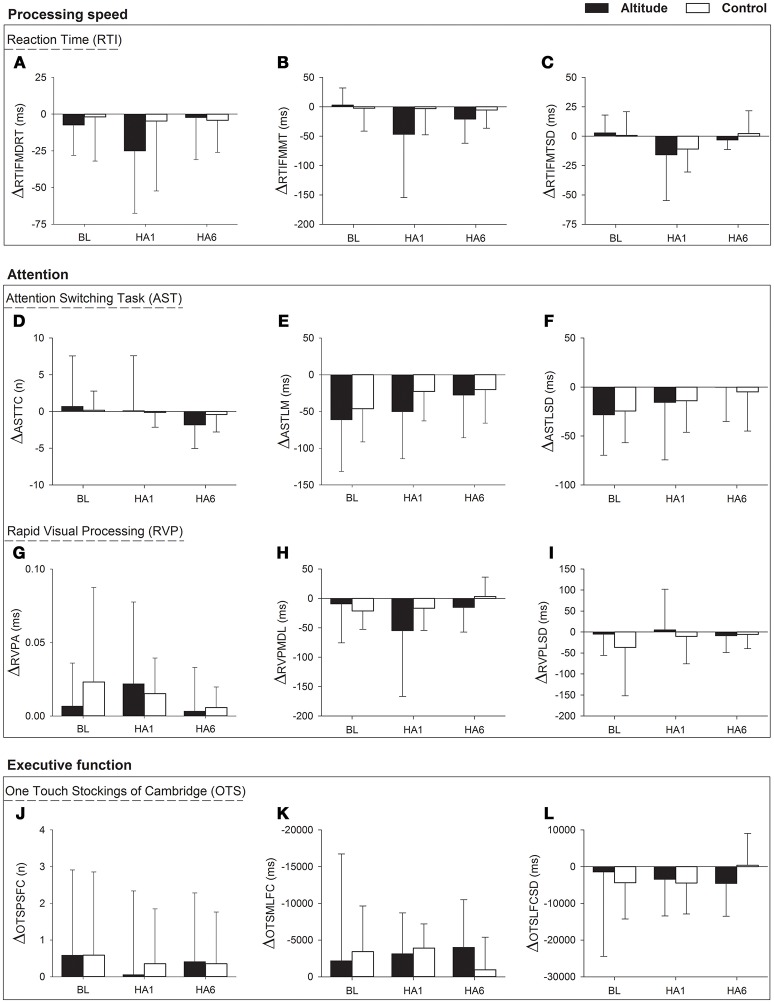
Changes in cognition (CANTAB outcome parameters with RM-ANOVA) over two cycles (ΔCANTAB = *Cycle 2* – *Cycle 1*) at very high altitude during acute, subacute and repeated exposure comparing with controls. Figure has three horizontal box panels. The first panel **(A–C)** illustrates “processing speed” i.e., changes in Reaction Time (RTI) parameters (ΔRTIFMDRT, ΔRTIFMMT, and ΔRTIFMTSD), the second box panel contains “attention” in which first panel within the box **(D–F)** shows changes in Attention Switching Task (AST) parameters (ΔASTTC, ΔASTLM, and ΔASTLSD) while the second panel within the second box **(G–I)** shows changes in Rapid Visual Processing (RVP) parameters (ΔRVPA, ΔRVPMDL, and ΔRVPLSD) and the third box panel **(J–L)** shows changes in One Touch Stockings of Cambridge (OTS) parameters (ΔOTSPSFC, ΔOTSMLFC, and ΔOTSLFCSD). The x-axis depicts different time points of data collection for Altitude exposure and Control groups at Baseline (BL), Acute exposure (HA1) and Acclimatization exposure (HA6). The y-axis depicts changes in cognitive parameters as mean ± SD for Altitude and Control group. Symbols: Black filled bars, Altitude group; empty bars, Control group; Δ, Change; ms, milliseconds; n, number. BL, baseline; HA1, acute exposure to altitude (day 1); HA6, acclimatization exposure to altitude (day 6); RTI, Reaction Time; AST, Attention Switching Task; RVP, Rapid Visual Processing; OTS, One Touch Stockings of Cambridge; ΔRTIFMDRT, RTI Median Five-choice Reaction Time; ΔRTIFMMT, RTI Mean Five-choice Movement Time; ΔRTIFMTSD, RTI Five-choice Movement Time Standard Deviation; ΔASTTC, AST Total Correct; ΔASTLM, AST Latency Mean; ΔASTLSD, AST Latency Standard Deviation; ΔRVPA, Rapid Visual Processing Accuracy; ΔRVPMDL, ΔRVP Mean Response Latency; ΔRVPLSD, RVP Response Latency Standard Deviation; ΔOTSPSFC, OTS Problems Solved on First Choice; ΔOTSMLFC, OTS Mean Latency First Choice; ΔOTSLFCSD, OTS Latency to First Choice Standard Deviation.

We explored the carry-over effect across the cycles (*Cycle 1* and *Cycle 2*) with the calculation of changes in cognitive functions i.e. ΔCANTAB = *Cycle 2 - Cycle 1* at BL, HA1 and HA6 time points. We then ran a RM-ANOVA on the cognitive outcomes change scores at ΔBL, ΔHA1. and ΔHA6. There was a main effect of altitude exposure on ASTLM [*F*_(2, 64)_ = 5.8, *p* = 0.005, $\eta_{p}^{2}$ = 0.154] with a smaller change in ASTLM over the acclimatization exposure compared to baseline [ΔBL (−61.2 ± 70.2) vs. HA6 (−28.0 ± 58.0, *p* = 0.007)]. Similarly, we found a main effect of altitude on ASTLSD score [*F*_(2, 64)_ = 4.0, *p* = 0.023, $\eta_{p}^{2}$ = 0.112] with a smaller change in ASTLSD over acclimatization exposure compared to baseline [ΔBL (−28.4 ± 41.2) vs. ΔHA6 (−0.2235 ± 34.8), *p* = 0.032]. For both outcomes (ASTLM and ASTLSD), there was no significant group by altitude exposure interaction. There were no significant changes across the two cycles for AST total correct nor other outcomes for the OTS, RVP, and RTI tests. The changes in cognitive function at altitude over two cycles as analyzed with RM-ANOVA have been illustrated in Figure 4.

We did not observe any significant differences in the percent changes of AST parameters during acclimatization indicating that the significant changes observed over the acclimatization period in *Cycle 1* are similar in magnitude as the changes observed in *Cycle 2*. Similarly, there were no significant differences in the percent changes at altitude from the baseline between the two cycles.

## Discussion

In this study, we investigated the effects of acute exposure, acclimatization and repeated exposure to very high altitude on cognitive functions in altitude-naïve individuals compared to control subjects tested at low altitude. We report four major findings. First, cognitive abilities, particularly sustained attention and inhibition of irrelevant information (selective attention) measured with the AST, significantly improved with acclimatization in both cycles. Second, the improvement gained in cognitive functions during the acclimatization period in *Cycle 1* did not carry over to the repeated exposure in *Cycle 2*. Third, changes in SpO_2_ explained changes in ASTLM score and ASTLSD score during acute and acclimatization exposures, but not during altitude stay in both cycles. Finally, the degree of acute mountain sickness, reflected by the LLS, explains in part the changes in AST scores (ASTLSD in *Cycle 1*, and ASTLM and ASTLSD in *Cycle 2*). We did not find any significant changes in reaction times, visual processing and executive functions during acute and acclimatization exposures. The novelty and strengths of this study include a strong experimental design, a robust cognitive battery not previously used in altitude studies and the use of a control group tested at low altitude.

Previous studies have used a variety of cognitive tests to separately assess processing speed, attention and executive functions (Harris et al., 2009; Subudhi et al., 2014; Nation et al., 2017; Phillips et al., 2017). However, none has used a battery to test these cognitive domains concurrently. Here, we used a custom cognitive battery built within the CANTAB (Cambridge Cognition, 1994; Strauss et al., 2006) cognitive test collection, which included tests to assess all three domains simultaneously. Custom batteries assembled within CANTAB have been shown to be robust and well suited for repeated measures testing (Syväoja et al., 2015). Within the analyses, we selected outcomes such as mean response latency, standard deviation, and accuracy for each cognitive domain, variables previously used and validated for neuropsychological assessments in other contexts and clinical populations (Sweeney et al., 2000). We included the RTI task because reaction times have been used as a measure of processing speed in both field (Kramer et al., 1993; Ma et al., 2015; Chen et al., 2017) and laboratory (hypoxic chamber) (Hornbein et al., 1989; Turner et al., 2015; Pramsohler et al., 2017) settings. Further, we chose the Attention Switching Task as a measure of attention and ability to inhibit irrelevant information. High altitude exposure significantly decreases test accuracy and increases reaction time in the “Word-Color Stroop Test,” a test that also measures inhibition and attention (Asmaro et al., 2013). Similarly, the choice of Rapid Visual Processing Task was based on the previous studies (Kramer et al., 1993; Finn and McDonald, 2012) in which this task was used due to its sensitivity to both neurological damage (Finn and McDonald, 2012) and high altitude exposure (Kramer et al., 1993). Finally, we chose the One Touch Stockings of Cambridge Test to assess executive functions at altitude in line with previous studies (Asmaro et al., 2013). Similarly, we used problems solved on first choice, Mean Latency and SD of first choice which are the outcomes that CANTAB recommends to assess planning and spatial working memory (Naef et al., 2017). To our knowledge, this is the first time that a CANTAB custom cognitive battery has been used to explore the effects of very high altitude and hypoxia exposure. Cognitive assessments in the altitude literature are often confounded by multiple factors such as mode and rate of ascent, absolute altitude gained, physical exertion or exercise, cold, radiation and individual susceptibility to hypobaric hypoxia (Virués-Ortega et al., 2004; Yan, 2014; Taylor et al., 2016; McMorris et al., 2017). In our study, we controlled these confounding factors by using a design that involved rapid ascent to very high altitude (5,050 m), minimal or no physical activity and lack of exposure to environmental stressors because participants remained inside the ALMA facility during the testing sessions.

### Processing speed

Previous studies have often reported a reduction in reaction times during acute exposure to altitude (Ma et al., 2015; Chen et al., 2017). It is often argued that the reduction in processing speed is a compensatory mechanisms to try to increase test accuracy at the expense of speed (Bahrke and Shukitt-Hale, 1993). Consistently with previous studies (Dykiert et al., 2010; Subudhi et al., 2014), we found that altitude exposure reduced the variability (SD) in the RTI Five-choice Movement Time but that the group-by-altitude interaction was not signficant in *Cycle 1*. During repeated exposure, in *Cycle 2*, altitude exposure had no effect on the RTI Five-time Movement time SD score although there was a group by altitude interaction (i.e., altitude and control subjects have different variances in processing speed at different altitudes). With acclimatization in *Cycle 2*, the RTI Five-time Movement time SD decreased during acclimatization although the significance was lost after *post-hoc* corrections. The lack of significant effects of acute exposure to altitude on reaction times, contrary to findings of other studies (Sharma et al., 1975; Dykiert et al., 2010), could be due to the small sample size and to the fact that participants were exposed to 5,050 m only 6–8 h/day and slept at lower altitude (2,900 m). Further, participants in our study may have benefitted from the specific exposure pattern with sleeping at lower altitude (Richalet et al., 2002; Farias et al., 2006; Vearrier and Greenberg, 2011) compared to other types of expedition/climbing exposure (Cavaletti et al., 1987; Kramer et al., 1993; Abraini et al., 1998). The mean and median five-choice reaction time scores did not vary significantly (neither the main effect of altitude nor the group-by-altitude interaction were significant) during acute, subacute and repeated altitude exposures. It is possible that the RTI task was too simple or not sensitive enough to measure the effects of high altitude exposure as reported previously (Roach et al., 2014). Alternatively, it is possible that complex reaction times are not profoundly affected below 6,000 m altitude (Virués-Ortega et al., 2004).

### Attention

In our study, we found that in the AST task both Mean Reaction Latency and Reaction Latency SD were significantly affected by altitude exposure and a group by altitude interaction (i.e., the altitude group and the control group performed differently with different altitude exposures with the only the altitude group showing differences at the three data points). AST Mean Reaction Latency score and AST Reaction Latency SD score improved over acclimatization compared to acute exposure but were not significantly different from baseline. This suggests that acclimatization plays a crucial role in restoring cognitive functions (Subudhi et al., 2014). The Mean Reaction Latency and SD seem to be more sensitive measures to assess the effects of high altitude exposure as compared to AST Total Correct score. With the repeated exposure in *Cycle 2*, in contrast to *Cycle 1*, there was a main effect of altitude on the AST Total Correct score and a group by altitude interaction. Particularly, the AST Total Correct score decreased during acute exposure and improved with acclimatization. In *Cycle* 2, both AST Mean Latency and AST Latency SD showed a main effect of altitude exposure as well as a group by altitude interaction. Both AST Mean Latency and AST Latency SD improved with acclimatization but only trended to increase (i.e., worse performance) with acute exposure. Interestingly, in both cycles, we did not find significant differences in AST Mean Latency and SD due to acute exposure but we found significant improvements with acclimatization. However, the observed changes did not translate to repeated exposures consistent with previous findings on re-ascent (Subudhi et al., 2014). The intriguing finding, i.e., no effect of acute exposure to altitude, may be due to the passive exposure (the ascent via motorized vehicles) and the lack of physical exertion at altitude. Our study participants were in fact comfortably resting at the ALMA Observatory facility at 5,050 m and measurements were taken a few hours after their arrival. Previous studies either recruited climbers (Cavaletti and Tredici, 1993; Kramer et al., 1993; Bonnon et al., 1999) and trekkers (Harris et al., 2009; Phillips et al., 2017) or simulated altitude by lowering the percentage of Oxygen (FiO_2_) and/or pressure in an altitude chamber (Hornbein et al., 1989; Pramsohler et al., 2017). This heterogeneity in the study population and absolute altitude reached makes it harder to compare findings from the different studies. The individual variability in cerebral hypoxia susceptibility during acute high altitude exposure (Cavaletti and Tredici, 1993) may also partially explain our findings. The consistent improvement over acclimatization in both cycles could be in fact due to our unique pattern of high altitude exposure (i.e., ~16 h spent at 2,900 m (sleeping altitude) and ~8 h spent at very high altitude (5,050 m). The pattern of repeated re-oxygenation (2/3 of the 24-h cycle spent at 2,900 vs. 5,050 m) with restful sleep might have significantly increased the beneficial effects of the acclimatization process and thereby improved AST outcomes. The testing schedule used in our study simulates the schedule of the workers at ALMA and other mining industries in the South American Andes (Richalet et al., 2002; Farias et al., 2006; Vearrier and Greenberg, 2011) and therefore differs slightly from the schedules commonly used in the field (Ma et al., 2015; Chen et al., 2017) or in chamber high altitude simulation (Hornbein et al., 1989; Turner et al., 2015). Overall our findings indicate that sustained attention and the ability to inhibit irrelevant information (selective attention) are impacted by acute high altitude exposure. This suggests that precision tasks that require long-term focus might be affected, and therefore, more difficult to execute during high altitude exposure.

We observed only subtle changes in the Rapid Visual Processing outcomes during altitude exposure in both *Cycle 1* and *2*. The significant effects observed were lost on *post-hoc* corrections for multiple comparisons. Our findings regarding RVP are consistent with findings from others who reported that rapid exposure to altitude has little effect on visual and auditory attention as compared to effects on learning and memory (Nation et al., 2017).

### Executive function

A previous study conducted in an altitude chamber with equivalent simulated altitude of 6,096 m (FiO_2_ = 10%) demonstrated impairments in cognitive functions including executive functions (Turner et al., 2015). We found a significant main effect of altitude exposure on both OTS Mean Latency to First Choice and SD scores but no group by altitude interaction. We did not find a significant main effect of altitude exposure on the OTS Problems Solved on First Choice score nor group by altitude interaction. Hence, the executive functions, measured with the OTS task, are not impaired by altitude exposure. Similar results were found for *Cycle 2*. The executive functions, as measured with the OTS task, do not seem to be affected by exposure to hypoxia as much as other cognitive domains (McMorris et al., 2017).

### Role of SpO_2_ and LLS score on cognition

#### SpO_2_ on cognitive changes

The cognitive changes following cerebral impairment due to altitude hypoxia could be related to changes in SpO_2_ (Yan et al., 2011; McMorris et al., 2017). In *Cycle 1*, the significant effects of altitude exposure disappeared when controlling for Acute SpO_2_ and Acclimatization SpO_2_ for AST Mean Reaction Latency scores and AST reaction Latency SD scores. Hence, the effects seen during acute and acclimatization exposures are primarily driven by hypobaric hypoxia. Interestingly, the significant result persisted when controlling for Altitude SpO_2_ in both AST Mean Reaction Latency scores and AST Latency SD scores. During the repeated exposure in *Cycle 2*, we found a similar pattern as in *Cycle 1*. Significant cognitive changes in the AST Mean Reaction Latency scores and AST Reaction Latency SD scores due to altitude exposure persisted when controlling for Altitude SpO_2_, but disappeared when controlling for Acute SpO_2_ and Acclimatization SpO_2_. The altitude effects on AST Mean Latency and AST Latency SD observed in the acclimatization period (HA6-HA1) in both cycles, provide strong evidence of a beneficial effect of acclimatization on cognition. On the other hand, the effects are cycle specific i.e., the effects found in *Cycle* 1 do not carry over to *Cycle 2*. Further, our results sheds light on the important role played by Altitude SpO_2_ (HA6-HA1) on cognitive functioning strengthening the idea of using oxygen supplementation at very high altitude to improve safety and work performance among scientists and workers (West, 2003, 2015; Moraga et al., 2018).

#### Total LLS score on cognitive changes

AMS symptoms are classified as cerebral symptoms (Wilson et al., 2009; Imray et al., 2010) and consequentially, they are expected to be associated with impaired cognitive functions at high altitude (Dykiert et al., 2010) although this relationship is still unclear (Virués-Ortega et al., 2004; Yan, 2014). In *Cycle 1*, the significant changes in AST mean reaction latency scores due to acute altitude exposure (HA1) persisted when including the LLS score as covariate in the model, indicating that the AMS symptoms are not associated with changes in AST Mean Reaction Latency scores. Conversely, the effect of altitude exposure on AST Reaction Latency SD score disappeared with LLS score entered as covariate, which indicates that AMS symptoms may have played a role in changes in cognitive abilities for this outcome. These findings suggest that AMS score could be associated with certain outcomes (AST Latency SD, an index of variability) but not others (AST Mean Latency) during acute altitude exposure. It is noteworthy that recent studies have found that poor sleep quality is not to be associated with AMS (MacInnis et al., 2013; Hall et al., 2014). It is therefore possible that some of the cognitive functions that are altered by sleep disturbances are not sensitive to high altitude exposure or symptoms of AMS. Consistently, Kramer and colleagues did not find any significant correlations between AMS severity and cognitive performance among climbers (Kramer et al., 1993). The rate of ascent, the absolute altitude gained and the physical activity in the field might be responsible for the discrepancy in the findings. With repeated exposure in *Cycle 2*, the cognitive changes related to acute altitude exposure (HA1) disappeared when using total LLS score as a covariate. The AMS symptoms seem to play a role in changes in cognitive abilities for these outcomes even during repeated exposures although the LLS score in the repeated exposure was significantly decreased compared to acute exposure (HA1, *Cycle 1*).

### Acclimatization, repeated exposure and carry-over effects

The principal reason behind the implementation of such work schedule in the Chilean workers with sleep periods at lower altitude, repeated exposure to 5,050 m, and a week of rest at sea-level is to try to minimize the adverse effects of very high altitude on workers' health and performance (Richalet et al., 2002; Farias et al., 2006; Vearrier and Greenberg, 2011) and allow them to see their families during the week of rest at low altitude. In our study which reproduces this schedule over two work-week cycles, we found a significant main effect of altitude exposure for the AST Mean Reaction Latency and AST Latency SD with a significant decrease in both AST Mean Reaction Latency and SD over the acclimatization exposure compared to baseline and AST Latency SD change across cycles. This indicates that the changes between baseline and acclimatization in *Cycle* 1 are different from the changes happening in *Cycle* 2, confirming that the significant improvement in cognitive functions with acclimatization is not maintained with a week of rest or with repeated exposure. Our findings are consistent with Subudhi and colleagues who reported that cognitive functions improved with acclimatization and the obtained gains are not completely retained upon re-ascent/repeated exposure (Subudhi et al., 2014). It is not entirely clear whether sleep at lower altitude favors this outcome or if this is a consequence of the acclimatization period itself. Further, we did not find any differences when comparing the two cycles in terms of percent change for AST during acute and acclimatization exposures. This means that the magnitude of the changes observed over the acclimatization in *Cycle 1* is not different from the magnitude of the changes observed in *Cycle 2*. Our findings suggest that the acclimatization of cognitive functions at altitude is a dynamic process and it may not reach a plateau within approximately a week, in contrast to other physiological variables such as ventilatory acclimatization (Pamenter and Powell, 2016). One possibility is that cognitive functions may not benefit from high altitude acclimatization (Taylor et al., 2016) unlike other physiological functions such as athletic performance. However, these relationships may be different at higher elevation, with longer durations of stay and increased physical exertion (Shukitt-Hale et al., 1998).

### Strengths and limitations

#### Strengths

We exposed young healthy altitude-naïve individuals to very high altitude following the same schedule as the ALMA workers. Thus, this study is highly relevant to a significant workforce in Chile and other parts of South America, and could be of interest to governments and policy makers who regulate work at high altitude. We used a custom cognitive battery generated from the Cambridge Neuropsychological Test Automated Battery (CANTAB) test collection, which has not been used previously in high altitude research. The comprehensive cognitive battery tests processing speed, attention and executive function was used to assess the effects of acute exposure, acclimatization exposure and repeated exposure to very high altitude. Participants completed the cognitive assessments at various time points of altitude exposure. Similarly, we recruited a control group at low altitude, to test the between-group differences as well as control for practice effects. The use of this custom testing battery constitutes a strength of the study. The neuropsychological assessment is in fact conducted using portable wireless touch screen tablets and therefore applicable in many remote settings. The data are then stored and can be either wireless transferred or downloaded later. Further we administered the battery in English but it is validated to be administered in multiple languages making it a very flexible assessment tool in a variety of populations and settings. Another strength of the study is the use of a experimental design that controls factors such as environmental stressors and the effects physical exertion. This design allowed us to untangle the effects of hypoxia at high altitude from other confounders.

#### Limitations

This study has also some limitations. First, it provides only a snapshot of the high-altitude exposure which is hard to compare to the repeated exposures of the high-altitude workers who have been following this schedule for many years. Second, we did not collect cognitive data at sleeping altitude i.e., mid-altitude (2,900 m) where participants (and workers) spend >15 h/day while they only spent ~8 h/day at 5,050 m. Sleeping at lower altitude and increased oxygen levels might have had beneficial effects that counteracted the negative effects of acute high-altitude exposure. These should be areas of further investigation. Third, the findings from the study should be interpreted carefully when generalizing to other types of high altitude exposure or paticipants' groups due to the unique ascent profile, relatively small sample size and the weekly shift-work schedule that was used in our study. Finally, the participants in this study were passively exposed to altitude (via travel by plane from 520 to 2,900 m and by motorized vehicle from 2,900 to 5,050 m) and had minimal physical exertion as opposed to previous studies in climbers and trekkers.

## Conclusions

The findings of our study highlight the importance of acclimatization on restoring cognitive function after acute exposure to very high altitude. However, it is important to consider that the gains in cognitive functions during the acclimatization period in the first exposure are not carried over to repeated exposures. The SpO_2_ is associated with cognitive changes during acute and acclimatization exposure and AMS scores might partially explain the cognitive changes. Taken together, our results suggest that the tasks that need sustained focus and high level of precision may be affected during acute exposure and also during repeated exposure or re-ascent. These findings and their implications for the safety and performance of the workers in the mine industry and other high-altitude workers highlight the importance of linking research groups and scientific findings with the organizational strategies of these specialized work sites. The findings would also be helpful for the related organizations and governments in policy formulation aiming at increasing the safety and security of high altitude workers. Future studies should focus on the effects of high altitude on learning and declarative memory, should include data collection at sleeping altitude (2,900 m) and most importantly, should recruit workers who have been working at high altitude for extended periods. The effects of room oxygen enrichment “oxygen conditioning” at high altitude (West, 2016a,b) for newcomers, as well as for high altitude residents, should also be the topic of future investigations.

## Ethics statement

The study was approved by the University of Calgary Conjoint Health Research Ethics Board (CHREB ID: REB15-2709).

## Author contributions

MP and VG organized the data, carried out the analyses, drafted the manuscript and took the lead of manuscript finalization and submission process. KMB, LD, and JA were involved in the control data collection, export, organization and preliminary analyses. KEB, BG, JR, and MJP were involved in the conceptualization, design, and planning of the study. SH, MF, SU, KEB, and MJP were involved in the field for the data collection, troubleshooting, and manuscript finalization. All authors went through all the versions of the manuscript and approved them.

### Conflict of interest statement

The authors declare that the research was conducted in the absence of any commercial or financial relationships that could be construed as a potential conflict of interest.
