# Supplementary material for: Effects on Cognitive Functioning of Acute, Subacute and Repeated Exposures to High Altitude
Source: Front Physiol. 2018 Aug 21;9:1131. doi: 10.3389/fphys.2018.01131 (PMC6111975; doi:10.3389/fphys.2018.01131)
Supplement: Supplementary file 1 [file Table_1.pdf]

Supplemental Table 1.

Table 1 (Supplemental). Absolute cognitive values during acute, subacute and repeated exposure at very high altitude among young altitude naïve individuals and controls at nine different time points of expedition

	Cycle 1										***Cycle 2***							
	**FL**		**BL**		**HA1**		**HA6**		**REC**		**BL**		**HA1**		**HA6**		**REC**	
	**Alt (Sant)**	**Ctrl (Calg)**	**Alt (Sant)**	**Ctrl (Calg)**	**Alt (ALMA)**	**Ctrl (Calg)**	**Alt (ALMA)**	**Ctrl (Calg)**	**Alt (Sant)**	**Ctrl (Calg)**	**Alt (Sant)**	**Ctrl (Calg)**	**Alt (ALMA)**	**Ctrl (Calg)**	**Alt (ALMA)**	**Ctrl (Calg)**	**Alt (Sant)**	**Ctrl (Calg)**
**Reaction time (ms)**																		
RTIFMDRT	370.2±34.8	380.4±59.0	365.7±39.0	356.8±34.5	387.9±75.6	354.9±49.1	360.4±36.3	352.8±30.8	354.1±34.7	353.8±29.0	361.4±38.0	354.9±32.9	356.8±33.4	350.2±29.4	351.0±41.6	348.7±26.5	352.5±38.2	343.7±25.6
RTIFMMT	209.2±60.5	191.9±35.8	189.5±74.7	189.9±44.1	261.8±178.1	188.6±43.3	207.8±80	190.5±41.5	188.0±73.1	191.3±44.2	194.5±84.7	187.5±38.9	200.0±74.4	185.4±39.5	175.8±61.9	184.9±41.0	172.1±63.7	180.7±38.4
RTIFMTSD	35.5±15.3	32.2±13.5	25.6±10.5	31.3±11.3	80.6±166.1	38.7±22.2	31.5±20.8	34.1±17.8	28.1±15.2	29.4±9.2	29.8±15.6	31.9±16.8	34.8±20.8	27.7±11.7	23.1±7.5	36.3±27.4	26.6±14.7	23.9±7.1
**Attention (ms/n)**																		
ASTTC (n)	155.8±2.9	155.4±8.1	155.2±7.5	157.8±2.7	150.4±15.1	157.2±2.6	154.2±10.7	157.4±2.3	156.2±3.6	157.0±2.3	156.0±2.9	158.0±2.5	152.4±7.2	157.1±2.5	154.8±3.7	156.9±3.3	155.0±6.1	158.1±3.0
ASTLM	622.8±105.1	588.4±85.5	568.8±96.4	516.0±83.6	584.1±91.3	494.6±77.0	525.8±91.2	490.8±78.8	508.9±91.9	482.5±79.4	506.8±77.2	469.9±67.8	523.3±97.1	471.9±70.2	485.2±82.1	470.7±63.9	479.5±90.5	453.9±60.6
ASTLSD	206.9±73.5	195.5±76.0	166.6±60.0	131.4±62.9	185.3±86.0	127.1±46.9	134.5±65.0	120.6±56.0	137.2±72.7	115.7±51.5	135.4±61.1	107.1±41.0	161.2±81.5	113.1±43.2	122.5±53.4	115.6±49.0	120.5±60.7	100.1±41.5
RVPA	0.94±0.05	0.94±0.07	0.96±0.04	0.96±0.07	0.94±0.07	0.97±0.03	0.97±0.04	0.98±0.02	0.97±0.04	0.98±0.02	0.97±0.05	0.98±0.02	0.96±0.05	0.99±0.02	0.98±0.04	0.99±0.02	0.98±0.04	0.99±0.01
RVPMDL	439.4±48.0	435.3±58.9	427.7±40.0	402.9±50.0	459.6±101.7	395.2±44.2	409.4±35.5	391.4±35.1	406.0±35.0	374.0±56.2	411.0±48.2	381.5±34.7	410.1±40.0	378.6±34.6	392.9±36.6	394.6±42.2	407.8±40.3	380.4±31.2
RVPLSD	158.7±84.7	154.8±145.0	111.4±42.5	117.8±123.0	133.2±101.5	95.8±63.4	108.9±75.1	91.4±47.0	80.2±47.6	80.7±48.0	101.7±57.3	81.2±44.0	131.1±93.0	85.3±40.0	88.8±44.7	85.6±56.6	91.6±43.8	75.6±39.7
**Executive function (ms/n)**																		
OTSPSFC (n)	11.8±2.7	11.8±1.1	12.3±1.8	12.2±1.8	11.9±1.7	12.5±0.9	12.1±2.0	12.4±1.5	12.8±1.5	12.8±1.5	12.9±1.7	12.8±1.9	12.1±2.1	12.9±1.9	12.6±1.7	12.8±1.6	12.0±2.3	13.4±0.9
OTSMLFC	16574.7±7369.1	18348.5±5250.5	14467.1±5520.0	13615.5±5282.7	12125.4±4984.4	14092.9±5766.3	10650.3±6909.1	10650.0±4035.9	9820.6±4590.9	10420.1±4205.4	11699.8±13680.1	10190.8±4799.3	8939.3±4922.7	10188.3±4683.9	6940.0±2141.0	9711.0±3933.1	7142.5±3129.5	9715.9±4481.1
OTSLFCSD	17992.0±14195.4	20871.6±8658.6	16042.8±8915.7	14867.7±8546.1	12826.8±7326.5	15925.1±10273.4	10191.9±8589.4	11074.2±6830.9	9684.7±6070.6	10691.1±6041.7	13632.4±20419.5	10490.0±7068.4	9440.9±7699.4	11474.9±7297.5	5990.2±2673.6	11422.2±7297.2	7048.1±5551.4	10624.9±6585.3

Table 1 (Supplemental). Descriptive CANTAB cognitive parameters of altitude and control participants from *Cycle 1* and *Cycle 2*

Note: The cognitive parameters (CANTAB outcomes) during acute, subacute and repeated exposure at very high altitude (ALMA, 5050m) among young altitude naive individuals. The controls underwent the testing with similar study design of data collection over nine consecutive sessions at their altitude of residence (Calgary, 1103m). The data presented are mean±SD from the descriptive statistics of all valid entry across the nine time points of high altitude exposure and controls

Abbreviations: FL, familiarization; BL, baseline; HA1, acute exposure to high altitude (day 1); HA6, acclimatization exposure to high altitude (day 6); REC, recovery; Alt, altitude; Ctrl, control; Sant, Santiago (520m); Calg, Calgary (1103m); ALMA, The Atacama Large Millimeter/submillimeter Array (5050m); ms, millisecond; n, number.

CANTAB parameter abbreviations: RTI, Reaction Time; AST, Attention Switching Task; RVP, Rapid Visual Processing; OTS, One Touch Stockings of Cambridge; RTIFMDRT, RTI Median Five-choice Reaction Time; RTIFMMT, RTI Mean Five-choice Movement Time; RTIFMTSD, RTI Five-choice Movement Time Standard Deviation; ASTTC, AST Total Correct; ASTLM, AST Latency Mean; ASTLSD, AST Latency Standard Deviation; RVPA, Rapid Visual Processing Accuracy; RVPMDL, RVP Mean Response Latency; RVPLSD, RVP Response Latency Standard Deviation; OTSPSFC, OTS Problems Solved on First Choice; OTSMLFC, OTS Mean Latency First Choice; OTSLFCSD, OTS Latency to First Choice Standard Deviation.
